# Editorial: Veterinary Dentistry and Oromaxillofacial Surgery in Wild and Exotic Animals

**DOI:** 10.3389/fvets.2022.871939

**Published:** 2022-03-11

**Authors:** Gerhard Steenkamp, Adrian S. W. Tordiffe, Ana Nemec

**Affiliations:** ^1^Department of Companion Animal Clinical Studies, Faculty of Veterinary Science, University of Pretoria, Pretoria, South Africa; ^2^Department of Paraclinical Sciences, Faculty of Veterinary Science, University of Pretoria, Pretoria, South Africa; ^3^Dentistry and Oral Surgery Department, Small Animal Clinic, Faculty of Veterinary Medicine, University of Ljubljana, Ljubljana, Slovenia

**Keywords:** wildlife, exotic animal, dentistry, skull, surgery

The human fascination with wild and exotic animals has existed for millennia (1). The first zoological garden in modern times was opened in 1752 in Vienna, Austria, and is still in existence today (2). A Google search for the current number of zoological gardens in the world shows in excess of 1,800 zoos (3). This does not include private collections, sanctuaries, orphanages, or rehabilitation centers. The Association for Zoos and Aquariums (AZA), based in North America, has 238 accredited zoos and aquariums in their membership located in 31 countries. It is estimated that they have more than 730,000 animals belonging to roughly 8,500 species (4). This diversity makes it difficult to accumulate detailed veterinary knowledge of each species in specialized fields like dentistry and maxillofacial surgery. As veterinary professionals, we need to strive for more evidence-based approaches in dealing with these patients and in veterinary dentistry we acknowledge this deficiency in our field. Veterinary dentistry in wild and exotic animals has been the topic of one previous Research Topic of articles in 2003, but since then no collective work has been published in this field (5).

In this Research Topic of five peer reviewed articles, two relate to clinical practice, two are skull-based studies, and the final study describes a new experimental model utilizing degus.

Since teeth and jaw bones have been preserved in museums for decades, this source of information is invaluable to the veterinary dentist who deals with wild and exotic species. Several well-constructed studies have been published on a variety of animals before (6–28). In fact, some of the most informative volumes of work were also based on museum collections (29–31).

Rickert et al. report on the temporomandibular joint (TMJ) pathology and the unique TMJ anatomy of wild carnivores in the western United States. They evaluated 5,011 animals belonging to 13 species and found TMJ osteoarthritis (TMJ-OA) to be the most common pathology present, possibly linked to masticatory forces. The authors call for ecological studies to describe the function of the TMJ during feeding and fighting in order to correlate function with the pathology seen.

The second article by Landy et al. utilized 28 skulls to describe the unique skull and dental pathology of an iconic Australia species, the Tasmanian devil (*Sarcophilus harrisii*). This study demonstrates the frustrations of retrospective studies utilizing skulls where metadata (e.g., sex, age, cause for death) are often absent and detracts from the ultimate value of such studies. The fact that a number of dental anomalies and pathologies were described on these wild animals is significant. It is the belief of the lead author that comparative studies between captive and wild specimens provide a deeper understanding of the pathological processes present in the captive environment and the opportunity to correct the poor husbandry practices that perpetuate such problems.

Clinical articles in the field of veterinary dentistry of wild and exotic animals are often limited to single animal case reports, while case series with good follow-up evaluations are far less common (32–34). The rarity of some of the species as well as the workload experienced by clinical veterinarians are factors that limit such publications.

The first clinical articles by Primožič et al. indicates the need for and describes the outcome of closed extractions of fractured canine teeth in 18 domestic ferrets (*Mustela putorius furo*). Although 50% of the fractured teeth had radiographic evidence of endodontal disease, poor correlation between pulpal health (as per histological examination) and radiographic evidence of endodontic disease was noted. The closed extraction, with extraction sites left to heal by second intention resulted in uncomplicated and rapid healing. At follow-up eight ferrets (44.4%) developed maxillary lip entrapment by the mandibular canine tooth, while two (11.1%) had mild lip trauma that did not require any further treatment.

The articles by Steenkamp et al. documented the presence and progression of a unique palatal pathology in 256 cheetahs (*Acinonyx jubatus*). Apart from this large cohort, a further five individuals were examined every 6 months from 7 months-of-age till 25 months-of-age. Cheetahs have deep indentation of the palatal mucosa where the mandibular premolar and molar teeth make contact with it. At the medial aspect of the maxillary fourth premolar tooth, there is a large indentation also in the palatine bone. These anatomical structures have previously been described (6) and is thought to be a function of the relatively large mandibular premolar and molar teeth. They introduce a clinical scoring system to quantify the defects which were observed over a period of 11 years. Based on the eruption patterns of the five cheetah cubs there was no super eruption of the mandibular molar teeth, nor was there any deviation of the teeth after they were fully erupted. This is in contrast with previous speculations. Inflammation of the depressions is often associated with food or debris trapped in them. Cleaning and flushing these depressions was enough to resolve the localized lesions and no molar tooth required an odontoplasty or extraction. This study further showed these lesions do not only occur caudally as previously reported, but can form in any of the depressions on the palate opposite mandibular premolar or molar teeth.

The final article in our Research Topic by Jekl et al. utilized the degu (*Octodon degus*) in an experimental study. The authors assessed the effect of chronic high dietary phosphorous levels on the mandibular ventral border, the volume of this bone apical to the fourth premolar tooth as well as the first and second mandibular molar teeth utilizing micro- computed tomography (micro-CT). Seventeen months after the start of the experiment, the degus on the high phosphorous diet had poor body condition scores, poor enamel covering the incisor teeth as well as irregular ventral mandibles with bony swellings. They also had a higher incidence of dental disease with elongation of the roots of the fourth premolar and first and second molar teeth. In addition, they had significantly thinner bone overlaying the apices of these teeth.

This Research Topic outlines the dilemma we have in wild and exotic dentistry today. We can produce good skull-based studies and case series, but evidence-based work is still scarce. Studies with larger cohorts should be encouraged through better collaboration of veterinary dentists globally. When dealing with wildlife, follow-up evaluations may indeed be difficult or impossible. The few cases where such good follow-up evaluations are available should be published to guide future work in this field.

## Author Contributions

GS drafted the editorial. AT and AN contributed to the editorial. GS, AT, and AN amended the editorial. All authors contributed to the article and approved the submitted version.

## Conflict of Interest

The authors declare that the research was conducted in the absence of any commercial or financial relationships that could be construed as a potential conflict of interest.

## Publisher's Note

All claims expressed in this article are solely those of the authors and do not necessarily represent those of their affiliated organizations, or those of the publisher, the editors and the reviewers. Any product that may be evaluated in this article, or claim that may be made by its manufacturer, is not guaranteed or endorsed by the publisher.
